# Serum Metabolomics Analysis for Biomarkers of *Lactobacillus plantarum* FRT4 in High-Fat Diet-Induced Obese Mice

**DOI:** 10.3390/foods11020184

**Published:** 2022-01-11

**Authors:** Hongying Cai, Zhiguo Wen, Xin Xu, Jiaxin Wang, Xuan Li, Kun Meng, Peilong Yang

**Affiliations:** 1 Key Laboratory of Feed Biotechnology of Ministry of Agriculture and Rural Affairs, Institute of Feed Research, Chinese Academy of Agricultural Sciences, Beijing 100081, China; caihongying@caas.cn (H.C.); wenzhiguo@caas.cn (Z.W.); 82101202006@caas.cn (X.X.); wjx13886114075@163.com (J.W.); lixuan01@caas.cn (X.L.); mengkun@caas.cn (K.M.); 2 National Engineering Research Center of Biological Feed, Beijing 100081, China

**Keywords:** obesity, *Lactobacillus plantarum* FRT4, metabolomics, biomarker, UHPLC-QTOF/MS

## Abstract

*Lactobacillus plantarum* is considered a potential probiotic supplementation for treating obesity. However, the underlying molecular mechanism is poorly understood. Our previous study displayed that *L. plantarum* FRT4 alleviated obesity in mice fed a high-fat diet (HFD) through ameliorating the HFD-induced gut microbiota dysbiosis. To explore the roles of FRT4 in obesity prevention, in this study, we investigated changes in serum metabolomic phenotype by ultra-performance liquid chromatography coupled with quadrupole time-of-flight mass spectrometry (UHPLC-QTOF/MS) and analyzed the pathway of HFD-fed Kunming female mice orally administered with FRT4 for eight weeks. Using orthogonal partial least squares discriminant analysis (OPLS-DA), metabolite patterns with significant changes were observed. 55 metabolites including phosphatidylcholine, lysophophatidylcholine, sphingomyelin, serotonin, indole-3-methyl aceta, indole-3-carbinol, indole-5,6-quino, 11,12-DHET, prostaglandin B2, leukotriene B4, and 3-hydroxybenzoic acid were identified as potential biomarkers associated with obesity, which were mainly involving in glycerophospholipid metabolism, tryptophan metabolism, and arachidonic acid metabolism. Perturbations of 14 biomarkers could be regulated by FRT4 intervention. These metabolites may serve as valuable biomarkers to understand the mechanisms by which intake of diets containing FRT4 contributes to the treatment or prevention of obesity. Thus, FRT4 can be a promising dietary supplement for the prevention of HFD-induced obesity.

## 1. Introduction

The availability of inexpensive, high-fat processed foods has contributed to the worldwide prevalence of obesity, which is one of the strongest risk factors for metabolic diseases, including type two diabetes mellitus (T2DM), hypertension, and cardiovascular disease [1]. The imbalance between energy intake and energy metabolism is the main cause of disease. Therefore, promoting metabolism through dietary intervention is a good approach to prevent obesity from the source and process [2].

Increasing studies have shown that probiotic treatment can ameliorate the disorders of host metabolism by regulating gut microbiota [3]. *Lactobacillus* is a major component of intestinal flora and is commonly used as a probiotic. In obese individuals, the supplementation of different strains of *Lactobacilli* has been shown to reduce fat mass while reducing the occurrence and progression of T2DM and insulin resistance [4]. *Lactobacillus plantarum* is one of the most well-studied species of *Lactobacillus* because of its versatility, allowing it to be distributed in a wide range of environments such as fermented foods and humans. Numerous studies have shown that *L. plantarum* has a variety of functional properties that exert beneficial effects on obesity. *L. plantarum* FRT10 had anti-obesity effects on obese mice, which was partly associated with activating PPARα/CPT1α pathway [5]. *L. plantarum* NCU116 alleviated non-alcoholic fatty liver disease (NAFLD) through the regulation of the expression of genes associated with lipogenesis, lipolysis, and fatty acid oxidation [6]. *L. plantarum* WCFS1 was found to increase the expression of immune response pathways [7] and epithelial tight junction proteins [8]. However, the mechanism by which probiotics alleviated obesity is not fully understood, and few studies have reported that probiotics improve the metabolomics of obesity caused by a high-fat diet (HFD). Metabolomics is a quantitative measurement of small molecular metabolites in biological samples, which provides a new perspective for studying the effects of diets or drugs by measuring the changes in metabolite levels and modeling them mathematically [9,10]. Metabolomics analysis techniques can detect dynamic changes in metabolite profiles in response to external stimulation [11]. Thus, metabolomics screening is considered a powerful tool for identifying biomarkers, providing extra information about the global metabolomic profiles of a complex biological system, and exploring new mechanisms. The accumulation of evidence associated with metabolic alterations is of great significance in guiding dietary interventions for the treatment of obesity with probiotics.

We previously established an HFD-induced obesity model in mice to study the regulatory effects of *L. plantarum* FRT4 on intestinal flora and liver metabolomics. The results suggested that FRT4 could effectively prevent obesity via alleviating HFD-induced gut microbiota dysbiosis, the mechanism of which could be partly revealed by liver metabolomics (data not shown). However, the exact mechanism is still unclear. Systemic metabolic disorders in patients can be reflected by serum markers, which are more suitable for clinical application because of their advantages of being non-invasive and at a low cost. Therefore, based on our previous studies, in this study, we further studied the effects of the dietary intervention of FRT4 on serum changes of HFD-induced mice during eight weeks of feeding via metabolomics method based on UPLC-QTOF/MS. Then, in order to find more specific obesity-related biomarkers and metabolic pathways, OPLS-DA analysis was used to determine the number and the distribution of metabolites with significant differences among each group. The aim of this follow-up research was to study the effects of FRT4 on serum metabolomics profile and provide more evidence for the potential mechanism of FRT4 against obesity. Meanwhile, this study may provide new insights into the pathogenesis of obesity, and the candidate biomarkers may be used for the diagnosis of obesity and related diseases.

## 2. Materials and Methods

### 2.1. Preparation of L. plantarum FRT4

*L. plantarum* FRT4 was isolated and identified from a kind of local yogurt in Xinjiang province and preserved in the Chinese General Microorganism Collection Center (accession 17956; CGMCC, Beijing, China). It was cultured in MRS broth at 37 °C for 24 h. After centrifugation at 5000 rpm at 4 °C for 10 min, the bacterial cells were collected, washed with 0.9% saline buffer three times, and adjusted to 1 × 10^10^ colony-forming units (CFU)/mL.

### 2.2. Animals and Grouping

Mice (Kunming female 7-week-old animals) were supplied by Vital River Laboratory Animal Technology Co. Ltd. (Beijing, China). After 1-week adaptation with the administration of a normal diet and water, the mice were randomly divided into two groups: control group (CT, n = 9) with a normal chow diet and HFD group (n = 18) fed a HFD based on the previous reports [5,12]. The mice were administered the HFD for 8 weeks and then were randomly assigned to two subgroups (9 mice in each group) and continuously administrated HFD. One served as the HFD control group (HF, n = 9), and the other received HFD and a daily oral dose of 0.2 mL FRT4 (HF4H, 1 × 10^10^ CFU/mL). All experiments were approved in accordance with the Laboratory Animal Ethical Committee and its Inspection procedures of the Institute of Feed Research of CAAS (AEC-CAAS-20090609).

### 2.3. Sample Collection

After 8 weeks of gavage treatment, all of the mice were sacrificed to collect blood after fasting for 6 h. The serum was obtained by centrifugation of the blood samples at 3000 rpm, 4 °C for 10 min and then was stored at −80 °C for metabolomics analysis.

### 2.4. Chemicals

Acetonitrile and methanol were supplied by CNW technologies. NH_4_AC and NH_4_OH were supplied by Sigma Aldrich and Fisher chemicals, respectively.

### 2.5. Metabolites Extraction

EP tubes containing 100 μL serum sample and 400 μL extract (methanol: acetonitrile = 1:1, *v*/*v*) were vortexed for 30 s, followed by ultrasonication in ice water bath for 10 min. After cooling down at −40 °C for 1 h, the mixtures were centrifuged at 12,000 rpm, 4 °C for 15 min. Supernatants of 400 μL were freeze dried, and the powders were solved in 150 μL methanol:acetonitrile (1:1, *v*/*v*) extract including isotope-labeled internal standard mixture, followed by vortex for 30 s and ultrasonication for 10 min. Then, the samples were centrifuged at 12,000 rpm at 4 °C for 15 min, and supernatants of the same amount from the whole samples were mixed into QC samples for further analysis.

### 2.6. UHPLC-QTOF MS Analysis

The UHPLC system (Vanquish, Thermo Fisher Scientific, Waltham, MA, USA) with a Waters ACQUITY UPLC BEH Amide column (1.7 μm × 2.1 mm × 100 mm) coupled to Q Exactive HFX mass spectrometer (Orbitrap MS, Thermo) was utilized to analyze serum metabolic profiling. The mobile phase consisted of 25 mM NH_4_Ac and 25 mM NH_4_OH (pH = 9.75) (A) and acetonitrile (B). The automatic injection temperature was maintained at 4 °C, and 3 μL was the injection volume.

On the basis of the information-dependent acquisition (IDA) mode, Xcalibur acquisition software (Thermo) was applied to acquire MS/MS spectra. The ESI source conditions were as follows: sheath gas flow rate, 30 Arb; Aux gas flow rate, 25 Arb; capillary temperature, 350 °C; full MS resolution, 60,000; MS/MS resolution, 7500; collision energy, 10/30/60 in NCE mode; spray voltage, 3600 V in positive ion mode, and 3200 V in negative ion mode.

### 2.7. UHPLC-QTOF MS Data Processing

The original data of MS were converted to the mzXML format by ProteoWizard and untargeted peak detection, peak extraction, peak alignment, and peak integration were processed by R package XCMS. Metabolites were identified using the in-house MS2 database (BiotreeDB). The cut-off value of the annotation was set to 0.3.

### 2.8. Statistical Analysis

After preprocessing the original data, OPLS-DA was applied to process the metabolomics analysis using SIMCA software (V15.0.2, Sartorius Stedim Data Analytics AB, Umea, Sweden). The fitness and reliability of the OPLS-DA were verified by the parameter values R^2^ and Q^2^. The permutation test of OPLS-DA was used to validate the model. The significantly differential metabolites were identified when variable importance for the projection (VIP) > 1 and *p* < 0.05 in the Student’s *t*-test.

Metabolites with significant differences (*p* < 0.05) were searched from the Kyoto Encyclopedia of Genes and Genomes (KEGG) database (www.genome.jp/kegg/, accessed on: 23 September 2020). Meanwhile, metabolites were retrieved from KEGG online database and corresponding metabolic pathways in KEGG were extracted. Compared with the model group, using differential abundance (DA) score was used to further screen the pathway affected by FRT4 intervention.

## 3. Results

### 3.1. Multivariate Statistical Analysis of Serum Metabolites

**Figure 1:**
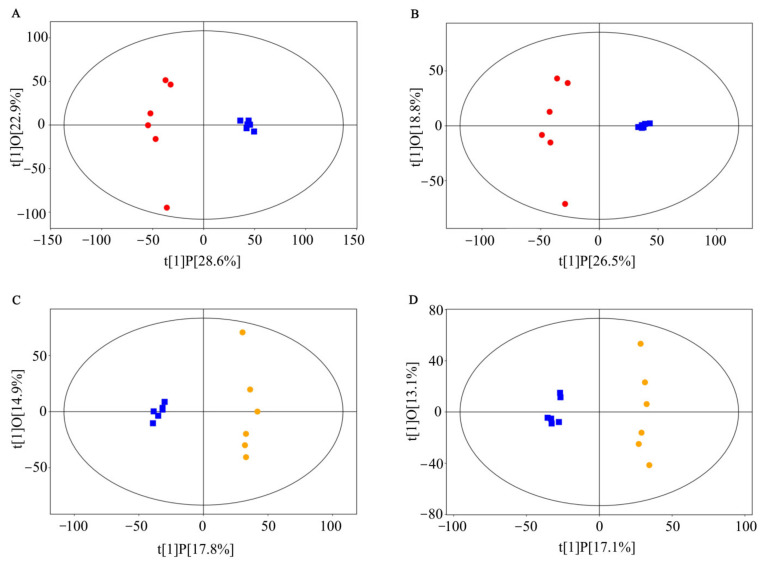
OPLS-DA score scatter plots of serum metabolites after *L. plantarum* FRT4 intervention. (**A**) HF versus CT group in ESI+ mode. (**B**) HF versus CT group in ESI- mode. (**C**) HF4H versus HF group in ESI+ mode. (**D**) HF4H versus HF group in ESI- mode.

The serum samples of mice were analyzed by UHPLC-QTOF/MS in the positive (ESI+) and negative ion (ESI-) modes. To reveal the differential metabolites among CT, HF and HF4H groups, the OPLS-DA model was used to determine the serum metabolic profile. As shown in Figure 1A,B, the OPLS-DA score plots showed clear clustering trends of metabolites between the HF and CT groups in both the ESI+ mode (R^2^X = 0.515, R^2^Y = 0.977,Q^2^ = 0.866) and the ESI- mode (R^2^X = 0.453, R^2^Y = 0.976, Q^2^ = 0.83), indicating significant changes in serum induced by HFD. OPLS-DA score plots revealed that there was a significant difference between the FRT4 group and the model group (Figure 1C,D). The parameters of OPLS-DA models including R^2^X = 0.327, R^2^Y = 0.989, Q^2^ = 0.533 in the ESI+ mode, and R^2^X = 0.302, R^2^Y = 0.99, Q^2^ = 0.564 in the ESI- mode. The results of OPLS-DA score plots suggested that FRT4 intervention partly recovered the serum changes caused by HFD.

**Figure 2:**
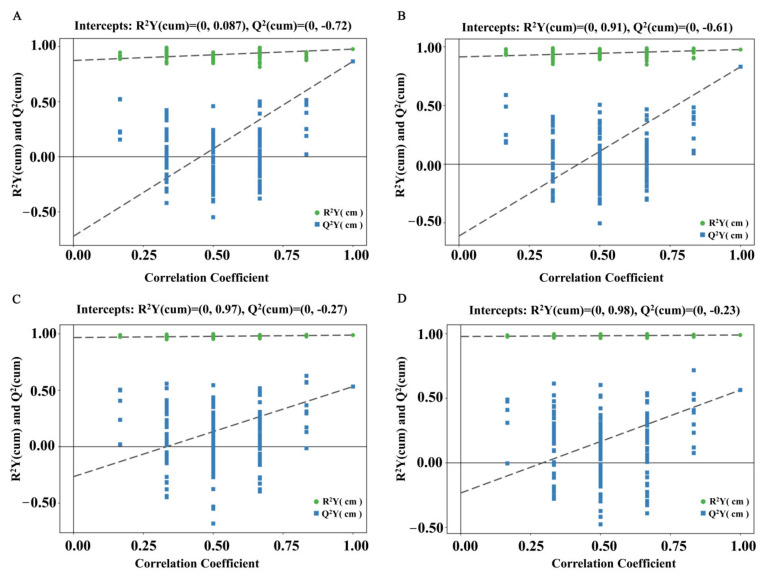
Permutation test displaying the stability of the OPLS-DA model. (**A**) HF versus CT in the ESI+ mode. (**B**) HF versus CT in the ESI- mode. (**C**) HF4H versus HF in the ESI+ mode. (**D**) HF4H versus HF in the ESI- mode.

As shown in Figure 2A,B, validation with 200 random permutation tests generated intercepts R^2^Y = 0.87, Q^2^ = −0.72 in the ESI+ mode, R^2^Y = 0.91, Q^2^ = −0.61 in the ESI- mode for HF versus CT group. As shown in Figure 2C,D, R^2^Y = 0.97, Q^2^ = −0.27 in the ESI+ mode, R^2^Y = 0.98, Q^2^ = −0.23 in the ESI- mode for HF4H versus HF group, which displayed that the OPLS-DA models had good robustness and there was no overfitting problem.

### 3.2. Metabolic Analysis of Serum Metabolites of FRT4 Treatment in Mice with HFD-Induced Obesity

According to VIP > 1 and *p* < 0.05, significantly differential metabolites at the superclass level were classified into 13 categories, including organoheterocyclic compounds; phenylpropanoids and polyketides; organic nitrogen compounds; organic acids and derivatives; nucleosides, nucleotides, and analogs; organic oxygen compounds; alkaloids and derivatives; organic compounds; lipids and lipid-like molecules; benzenoids; nucleosides, nucleotides, and analogs; lignans, neolignans, and related compounds; and hydrocarbons. Finally, 55 differently abundant metabolites were identified (Table S1). Compared to the CT group, HFD significantly altered the relative strength of a number of potential biomarkers involved in glycerophospholipid metabolism, tryptophan metabolism, phenylalanine metabolism, phenylalanine, tyrosine and tryptophan biosynthesis, and arachidonic acid metabolism. Biomarkers like glycerophosphocholine, phosphatidylcholine (PC), lysophosphatidylcholine (LysoPC), sphingomyelin (SM), serotonin, indole-3-methyl aceta, indoleacetaldehyde, and 8-HETE were significantly increased (*p* < 0.05), while kynurenic acid, 3-methyldioxyindole, indole-3-carbinol, indole-5,6-quino, 11,12-DHET, prostaglandin B2, and leukotriene B4 were significantly decreased (*p* < 0.05). Notably, FRT4 intervention partly reversed the abnormal serum metabolites alterations caused by HFD.

The serum metabolites of HF group were significantly different from those in CT group. As shown in Table S1, glycerophosphocholine and twenty PCs including PC(22:6(4Z,7Z,10Z,13Z,16Z,19Z)/18:0), PC(22:6(4Z,7Z,10Z,13Z,16Z,19Z)/18:0), PC(22:5(7Z,10Z,13Z,16Z,19Z)/18:2(9Z,12Z)), PC(20:3(5Z,8Z,11Z)/P-18:0), PC(22:6(4Z,7Z,10Z,13Z,16Z,19Z)/20:3(5Z,8Z,11Z)), PC(20:4(8Z,11Z,14Z,17Z)/P-18:0), PC(18:1(11Z)/14:0), PC(20:5(5Z,8Z,11Z,14Z,17Z)/P-18:0), PC(18:2(9Z,12Z)/P-18:1(11Z)), PC(22:5(4Z,7Z,10Z,13Z,16Z)/P-18:0), PC(22:5(7Z,10Z,13Z,16Z,19Z)/16:1(9Z)), PC(P-18:1(11Z)/22:5(4Z,7Z,10Z,13Z,16Z)), PC(20:3(8Z,11Z,14Z)/20:1(11Z)), PC(18:3(6Z,9Z,12Z)/18:0), PC(22:5(4Z,7Z,10Z,13Z,16Z)/20:5(5Z,8Z,11Z,14Z,17Z)), PC(22:6(4Z,7Z,10Z,13Z,16Z,19Z)/22:6(4Z,7Z,10Z,13Z,16Z,19Z)), PC(22:6(4Z,7Z,10Z,13Z,16Z,19Z)/20:2(11Z,14Z)), PC(18:0/P-16:0), PC(22:5(7Z,10Z,13Z,16Z,19Z)/20:1(11Z)), and PC(18:1(11Z)/P-16:0) were significantly increased and three PCs including PC(22:6(4Z,7Z,10Z,13Z,16Z,19Z)/18:3(6Z,9Z,12Z)), PC(22:4(7Z,10Z,13Z,16Z)/16:0), and PC(18:4(6Z,9Z,12Z,15Z)/18:1(11Z)) were significantly decreased, while FRT4 administration led a significant decrease of PC(20:3(5Z,8Z,11Z)/P-18:0), PC(20:3(5Z,8Z,11Z)/P-18:0), PC(16:0/P-16:0), PC(22:2(13Z,16Z)/15:0), PC(20:2(11Z,14Z)/15:0), and PC(15:0/15:0). In consistent with the stimulation of PCs in serum, the levels of LysoPCs including LysoPC(P-18:1(9Z)), LysoPC(22:4(7Z,10Z,13Z,16Z)), LysoPC(22:1(13Z)), and LysoPEs including LysoPE(18:3(6Z,9Z,12Z)/0:0), LysoPE(20:4(5Z,8Z,11Z,14Z)/0:0), and LysoPE(0:0/18:2(9Z,12Z)) were significantly increased in obese mice, while FRT4 intervention significantly reduced the level of LysoPC(22:1(13Z)). In addition, four SMs including SM(d18:1/16:0), SM(d18:0/18:1(9Z)), SM(d18:1/18:1(9Z)), and SM(d18:1/14:0) were significant increased, and SM(d17:1/24:1(15Z)) was significantly decreased, while FRT4 treatment significantly reduced the level of SM(d18:1/16:0), SM(d18:1/16:0), SM(d16:1/24:1(15Z)), and SM(d18:1/20:0). Furthermore, FRT4 treatment significantly reduced choline, PI(18:1(9Z)/18:1(9Z)), and PE(P-18:1(11Z)/18:4(6Z,9Z,12Z,15Z)) level.

Additionally, 8 potential biomarkers associated with tryptophan metabolism were also identified, including serotonin, indole-3-methy aceta, indole-3-carbinol, indole-5,6-quino, indoleacetaldehyde, 5-methoxytrytophan, and N-methynicotinamide. The relative content of serotonin, indole-3-methy aceta, indoleacetaldehyde, and 5-methoxytrytophan increased significantly in HFD-fed mice compared to the CT group, while decreasing significantly after FRT4 intervention. In contrast, indole-3-carbinol, and indole-5,6-quino were significantly reduced, while FRT4 intervention showed recovery patterns for these metabolites. Moreover, FRT4 intervention significantly reduced the level of indoleacetaldehyde, indole, 3-indoleacetonitrile, and 5-hydroxyindoleacetic acid. The results displayed that tryptophan metabolism disorder was generated by HFD, whereas FRT4 treatment reversed this trend, which indicated that the effects of FRT4 on tryptophan metabolism might be one of the reasons for its obese efficacy 11,12-DHET, prostaglandin B2, leukotriene B4, 15-deoxy-d-12,14-PGJ2, 5-KETE, prostaglandin G2, and prostaglandin D2 involved in arachidonic acid metabolism were decreased 0.084, 0.36, 0.22, 0.071, 0.22, 0.0077, and 0.097-fold, respectively, while FRT4 treatment increased 11,12-DHET, prostaglandin B2, leukotriene B4, and 15-deoxy-d-12,14-PGJ2 2.65, 2.23, 2.17, and 44.78-fold, respectively. Besides, FRT4 treatment increased 8-isoprostaglandin F2a (2.01-fold) and lipoxin B4 (1.69-fold). These alterations were significantly prevented after FRT4 intervention, suggesting that the favorable effect of FRT4 on obesity might be associated with regulating arachidonic acid metabolism.

Furthermore, three potential biomarkers related to phenylalanine metabolism were found in the HF group, including hippuric acid, 2-phenylacetamide, and L-tyrosine, which were significantly lower than those in CT group, while hippuric acid and 2-phenylacetamide were significantly reduced after FRT4 intervention. Finally, 3-hydroxybenzoic acid and 4-hydroxybenzoic acid associated with phenylalanine, tyrosine, and tryptophan biosynthesis metabolism were significantly decreased compared to the CT group, while FRT4 significantly increased 3-hydroxybenzoic acid.

### 3.3. Metabolic Pathway Affected by FRT4 Intervention

**Figure 3:**
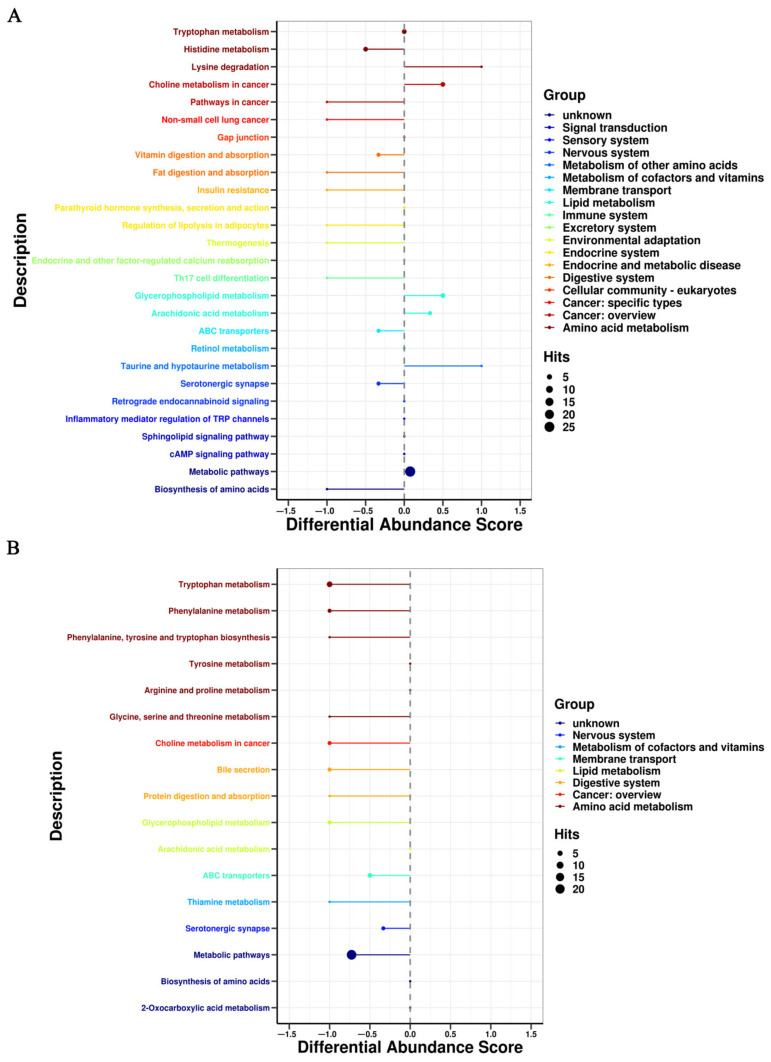
The disturbed pathway analysis using DA score in the ESI+ mode. (**A**) HF versus CT group. (**B**) HF4H versus HF group.

**Figure 4:**
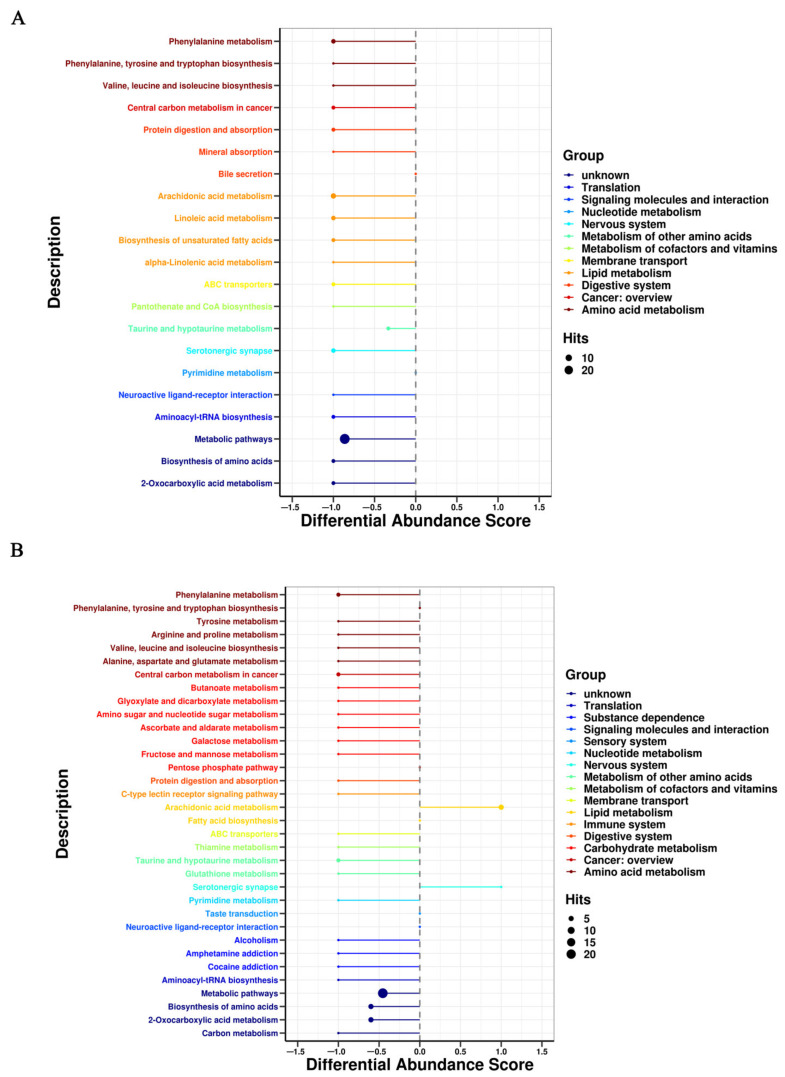
The disturbed pathway analysis using DA score in the ESI- mode. (**A**) HF versus CT group. (**B**) HF4H versus HF group.

**Figure 5:**
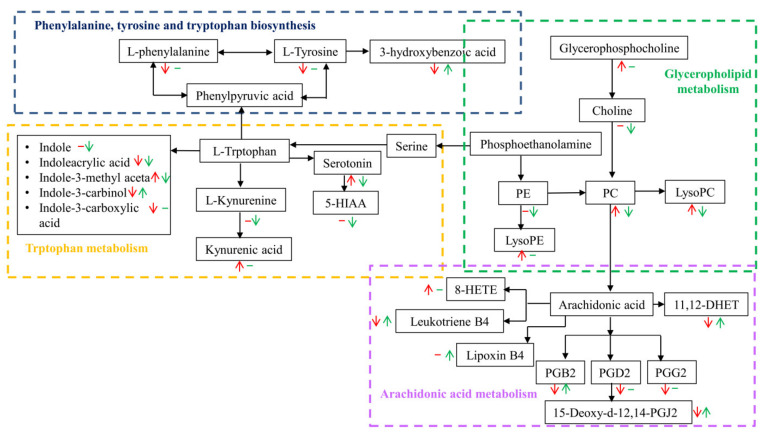
Manually linked metabolic pathway map based on the KEGG database involving glycerophospholipid metabolism, tryptophan metabolism, arachidonic acid metabolism, and phenylalanine, tyrosine, and tryptophan biosynthesis metabolism. Beneath each metabolite, red represents HF versus CT group and green represents HF4H versus HF group. The up arrows showed the metabolites were up-regulated and the down arrows showed the metabolites were down-regulated. Horizontal lines showed no significant difference in metabolites.

On the basis of *p* < 0.05 and pathway impact value, metabolic pathways with a significant difference in response to FRT4 treatment in obese mice were evaluated by KEGG annotation and pathway-based differential abundance (DA) analysis. As shown in Figure 3 and Figure 4, compared with the CT group, consistent with the differences identified in serum, lipid metabolism including glycerophospholipid metabolism and arachidonic acid metabolism were up-regulated and had higher DA scores in HF group, while were down-regulated and had lower DA scores after FRT4 intervention. Moreover, tryphotophan metabolism, phenylalanine metabolism, phenylalanine, tyrosine, and tryptophan biosynthesis metabolism were also down-regulated after FRT4 intervention. Thus, FRT4 intervention could partly reverse the alterations caused by the HFD. A comprehensive view of metabolic pathway analysis was shown in Figure 5.

## 4. Discussion

Our previous report has successfully established the obese model in mice with metabolic disorders in cecum contents and liver tissue after 8 weeks of continuous treatment of HFD [13]. Supplementation with *L. plantarum* FRT4 could alleviate HFD-induced obesity in mice through modulating gut microbiota and liver metabolomics (data not shown). In this study, analysis of the serum metabolites profile by UHPLC-QTOF MS indicated that the mice fed HFD were abnormal in serum, mainly involved in glycerophospholipid metabolism, tryptophan metabolism, arachidonic acid metabolism, phenylalanine metabolism, and phenylalanine, tyrosine, and tryptophan biosynthesis metabolism. In addition, FRT4 intervention had an inhibitory effect on obesity, which might be related to the recovery of some biomarkers.

The disfunction of glycerophospholipid and fatty acid metabolism has been identified to be directly associated with hyperlipidemia. Glycerophospholipid levels in the HF group were higher than that in the CT group, suggesting that HFD caused lipid disorders. In accordance with our previous report, most glycerophospholipids in the liver of obese mice were significantly enhanced [13], and the accumulation of glycerophospholipids in serum was also found in this study. Twenty increased PCs and two decreased PCs corresponded to a previous study that most PCs in serum were significantly enhanced [14], and the increased levels of plasma PC were positively associated with fat accumulation [15]. Most of the excess PC synthesized in these mice is subsequently decomposed into diacylglycerol (DAG), which was re-esterified into TG [16,17]. However, the result is not in accordance with that PC treatment can alleviate fat accumulation [18,19]. In the membranes of all mammalian cells, PC and PE are the two most abundant phospholipids, which are mainly distributed in the outer and inner leaflets of the plasma membrane bilayer, respectively [20,21]. Ulcerative colitis is a chronic inflammatory state of the distal ileum and colon, which are characterized by decreased content of PC in the mucus layer [22]. Research has shown that the fluidity and permeability of cell membranes could be changed by fatty acids in response to external stress and the membrane could be protected by inhibition of fatty acid degradation [23]. It indicated that HFD increased glycerophospholipid metabolism through the catabolism of PCs and destroyed the membrane. The significantly decreased PCs (*p* < 0.05) after FRT4 intervention suggested that the administration of FRT4 suppressed PC degradation and protected the membrane. Phosphatidylethanolamine (PE) is a class of phospholipids that exist on the cell membrane of all living organisms and is the backbone of most biological membranes along with PC, phosphatidylserine (PS), and phosphatidylinositol (PI). In this study, PI(18:1(9Z)/18:1(9Z)) and PE(P-18:1(11Z)/18:4(6Z,9Z,12Z,15Z)) were decreased after FRT4 intervention. Consistent with the stimulation of PCs, LysoPCs and LysoPEs, which were products or metabolites PCs and PEs, respectively, were significantly enhanced in the HF group as compared to the CT group. Altered levels of LysoPCs are identified to be related to diseases with the disorder of energy, such as obesity and hyperlipidemia [24]. LysoPC(18:1(9Z)) was increased after HFD feeding compared with those in the CT group, which is in accordance with increased LysoPC(18:1(9Z)) in HFD-fed rats [25]. LysoPC(22:4(7Z,10Z,13Z,16Z)), LysoPC(22:1(13Z)), LysoPE(18:3(6Z,9Z,12Z)/0:0), LysoPE(18:3(6Z,9Z,12Z)/0:0), and LysoPE(0:0/18:2(9Z,12Z)) were enhanced in HF group, suggesting the glycerophospholipid metabolism was promoted under hyperlipidemia condition. It was partially matched with other reports that plasma levels of LysoPC (C14:0), LysoPC (C18:0) were significantly higher in overweight than in lean subjects [26]. FRT4 intervention showed favorable regulation on the variation of LysoPC(22:1(13Z)), and there was no significant difference of LysoPE between FRT4 and HFD-fed groups. Moreover, glycerophosphocholine was also significantly up-regulated in obese mice and FRT4 treatment significantly down-regulated choline. Glycerophosphocholine was metabolized to choline, which acted as a substrate to resynthesize PC [27]. Choline plays an important role in the mitochondrial membrane and the disfunction of mitochondria is a critical mechanism of NAFLD [28]. Choline was down-regulated, which explained the lower level of PCs after FRT4 treatment. However, it was not in accordance with the study that choline was significantly enhanced in serum after Lp supplementation [29]. These metabolism alterations in PCs, LysoPCs, and LysoPEs contribute to further understanding the underlying mechanism of FRT4 intervention. In addition to glycerophospholipids, some sphingolipids have been found to play potential roles in the development of NAFLD [30,31]. Sphingolipid metabolism plays an important part in regulating inflammatory signaling pathways, and dietary sphingolipids seem to affect inflammation-related chronic diseases by changing gut microbiota [32]. Our results displayed that some SM species were significantly altered after HFD feeding, which was consistent with other studies [33,34]. Moreover, we found that FRT4 intervention showed a lower level of SM and reversed the abnormal level of SM (d18:1/16:0) compared with the HFD group. This result was inconsistent with the previous report that BL21 showed a higher hepatic level of SM [23]. These findings may suggest that different sphingolipids with specific functions still need further clarification. Our results also suggested that the mice treated with FRT4 protected the cell membrane by reshaping glycerophospholipid metabolism. However, the alterations in the cell membrane by which metabolites are not well understood, and this interaction needs to be elucidated in future studies.

Obesity is a complex pathophysiological condition characterized by chronic low-grade inflammation and alterations in the gut microbial ecosystem. Marques et al. reported that inflammation was related to altered metabolism of tryptophan (Trp), the most restricted essential amino acids [3] and precursor of serotonin, kynurenine (KYN), and indoles. In the present study, a high-fat diet impaired tryptophan metabolism. Serotonin is a monoamine neurotransmitter derived from tryptophan that is involved in appetite regulation. Accumulating evidence indicates that serotonin could inhibit food intake and body weight gain [35,36]. HFD-fed mice had significantly higher serotonin levels compared to the normal mice, which is in line with the study showing that HFD-fed mice had much higher serotonin blood levels than lean mice [15]. The result contradicted the reports that plasma level of serotonin was significantly decreased in obese subjects [37], and the decrease of circulating serotonin levels in patients with metabolic syndrome was negatively correlated with BMI and body fat [38]. FRT4 intervention significantly reduced serotonin and 5-hydroxyindoleacetic acid (5-HIAA). 5-HIAA is one of the metabolites of serotonin. Serotonin is one of the most essential degradation products of Trp pathway and is closely related to the development of cardiovascular disease [39]. Indoleacrylic acid, involved in serotonin pathway, was significantly reduced in the study. Indoleacrylic acid affects the content of unsaturated fatty acids in the membrane by phospholipases activity. The data is in line with the study that the low level of indoleacrylic acid represented disturbances in cell membrane permeability [40]. Compared to the CT group mice, kynurenic acid (KA) was significantly enhanced, and indole-5,6-quino was significantly reduced in the HF group. KA is a metabolite of tryptophan and is degraded mainly through the kynurenine (KYN) pathway [41]. KA plays a mucosa protective and immunoregulatory role possibly through its G protein-coupled receptor GPR35, which is mainly expressed in epithelial and immune cells [42]. The data is in line with decreased KA levels in the serum of obese subjects compared with healthy ones [43]. There is no significant difference of KA between the FRT4 treatment group and HFD-fed mice, but FRT4 reversed indole-5,6-quino to some extent. Furthermore, the indole tryptophan metabolites by gut microbiota of indole-3-carbinol, indole-3-carboxylic acid, and indolelactic acid were significantly decreased in the HFD-fed group, which is in line with evidence that obesity is related to a significant decrease in microbial-derived indoles [38]. These metabolites might be responsible for the anti-inflammatory and favorable metabolic effects of the gut microbiota [44]. FRT4 intervention enhanced indole-3-carbinol and continuously reduced indolelactic acid. Interestingly, FRT4 intervention significantly reduced indole. Bacterial indole produced by dietary tryptophan decreased TNFα-mediated NF-kB activation and IL-8 expression while promoting anti-inflammatory IL-10 production and enhancing tight junction resistance in HCT-8 intestinal epithelial cells [45]. Fecal indole concentration in the lower-fat diet group was decreased, while that in the higher-fat diet group was increased [46]. Indole, the precursor of indoxyl sulfate, is associated with cardiovascular disease in patients with hypertension and chronic kidney disease [47]. Our results indicated that tryptophan metabolism was down-regulated after FRT4 intervention. Further research is required to investigate the beneficial effect of FRT4 on tryptophan metabolism in obese mice.

Arachidonic acid (AA) and its derivatives play a critical role in the development of obesity, NAFLD, diabetes, cardiovascular disease, and other common diseases by linking nutrient metabolism with immunity and inflammation [48]. 11,12-DHET, prostaglandin B2 (PGB2), prostaglandin D2 (PGD2), prostaglandin G2 (PGG2), leukotriene B4, 15-Deoxy-d-12,14-PGJ2, and 5-KETE involved in arachidonic acid metabolism were significantly decreased, and FRT4 treatment significantly increased their relative content. These substances come from long-chain polyunsaturated fatty acids and act as mediators of the intensity and duration of inflammation [49]. PGs, leukotrienes, and HETEs are pro-inflammatory AA metabolites [48]. Interestingly, PGB2, PGD2, and PGG2 were decreased, while 8-HETE was significantly increased in HFD-fed mice. The result is not in good agreement with the study that the arachidonic acid metabolism pathway was elevated in response to the higher-fat diet [46]. This could be explained by the fact that endogenous AA is produced mainly through the release of AA from cell membrane phospholipids. As we discussed above, HFD induced the enhanced catabolism of glycerophospholipid metabolism and thus increased the permeability of cell membranes. The host attempted to maintain phospholipid homeostasis of the mucus layer and thus reduced the release of AA from phospholipids. The group treated with FRT4 showed recovery patterns for PGB2, PGD2, and PGG2. In contrast, lipoxins have been found to ameliorate insulin sensitivity and possibly inhibit the occurrence and progression of diabetes mellitus [50]. Lipoxin B4 as anti-inflammatory AA metabolites was significantly enhanced after FRT4 treatment. The result indicated that FRT4 could ameliorate the disturbed arachidonic acid metabolism.

Although we found these findings in this research, limitations to understanding the preventive effects of FRT4 on metabolic dysfunction remain. Therefore, further investigations are needed to characterize the metabolites, elucidate the specific functions of individual metabolites and the preventative effects of FRT4 on related metabolic pathways.

## 5. Conclusions

The present study suggested that FRT4 intervention had a favorable inhibition effect on obesity, which might be related to the efficacy of FRT4 by restoring some of the identified biomarkers. 14 identified potential biomarkers in serum associated with the disturbance of the metabolism could be regulated by FRT4 in obese mice. FRT4 intervention showed partial recovery patterns for PCs, LysoPCs, SMs, PGs, and serotonin involving glycerophospholipid metabolism, tryptophan metabolism, and arachidonic acid metabolism, which were thought to be central factors in the prevention of obesity. Our results also predicted that the FRT4-treated mice could protect the cell membrane by remodeling glycerophospholipid metabolism and arachidonic acid metabolism. These metabolites could provide the basis for further understanding the pathogenesis of obesity and HFD-related diseases, increase the predictability of obesity risk, and evaluate the efficacy of anti-obesity drugs. These results support the concept that FRT4 had beneficial effects in HFD-induced obesity-associated metabolic disorders. These findings demonstrated the effects of FRT4 on serum metabolomics profile, which could provide new evidence for understanding the potential mechanism of FRT4 in preventing obesity.

## Data Availability

The data presented in this study are available within the article.

## Supplementary Materials

The following supporting information can be downloaded at: www.mdpi.com/article/10.3390/foods11020184/s1, Table S1. Differentially expressed endogenous metabolites detected by UHPLC-QTOF/MS among CT, HF, and HF4H groups.
