# Supplementary material for: Serum Metabolomics Analysis for Biomarkers of Lactobacillus plantarum FRT4 in High-Fat Diet-Induced Obese Mice
Source: Foods. 2022 Jan 11;11(2):184. doi: 10.3390/foods11020184 (PMC8774460; doi:10.3390/foods11020184)
Supplement: Supplementary file 1 [file foods-11-00184-s001.zip › foods-1494052-supplementary.pdf]

foods

Table S1. Differentially expressed endogenous metabolites detected by UHPLC-QTOF/MS among CT, HF, and HF4H groups.

			HF vs CT			HF4H vs HF		
RT(min)	m/z	Metabolites	VIP	FC	*p* value	VIP	FC	*p* value
433.6005	104.1074994	Choline				1.48	0.77	0.035
483.935	258.109992	Glycerophosphocholine	1.44	1.89	0.00019			
38.1646	834.59684682 6732	PC(22:6(4Z,7Z,10Z,13Z,16Z,19Z)/18:0)	1.32	2.16	0.0063			
168.248	832.5813204	PC(22:5(7Z,10Z,13Z,16Z,19Z)/18:2(9Z,12Z))	1.29	1.76	0.023			
168.2375	796.6173136	PC(20:3(5Z,8Z,11Z)/P-18:0)	1.51	5.02	1.92E-05	1.57	0.72	0.036
194.452	828.5393395	PC(22:6(4Z,7Z,10Z,13Z,16Z,19Z)/18:3(6Z,9Z,12Z))	1.50	0.011	0.045			
166.495	856.5800667	PC(22:6(4Z,7Z,10Z,13Z,16Z,19Z)/20:3(5Z,8Z,11Z))	1.39	1.77	0.011			
38.1378	794.6052084	PC(20:4(8Z,11Z,14Z,17Z)/P-18:0)	1.69	3.09	0.00052			
177.4725	732.5534861	PC(18:1(11Z)/14:0)	1.39	1.58	0.0092			
168.278	792.5886872	PC(20:5(5Z,8Z,11Z,14Z,17Z)/P-18:0)	1.60	3.54	0.00023			
38.1675	768.5870183	PC(18:2(9Z,12Z)/P-18:1(11Z))	1.38	2.01	0.011			
58.85805	806.5699089	PC(22:5(7Z,10Z,13Z,16Z,19Z)/16:1(9Z))	1.36	1.82	0.0078			
38.17195	818.6054727	PC(P-18:1(11Z)/22:5(4Z,7Z,10Z,13Z,16Z))	1.27	1.67	0.020			
168.2835	838.6258361	PC(20:3(8Z,11Z,14Z)/20:1(11Z))	1.39	2.27	0.0035			
90.34105	810.6002569	PC(22:4(7Z,10Z,13Z,16Z)/16:0)	1.67	5.60	0.0080			
180.3475	816.5734736	PC(P-18:1(11Z)/22:6(4Z,7Z,10Z,13Z,16Z,19Z))	1.40	0.02	0.029			
173.1085	784.5830106	PC(18:3(6Z,9Z,12Z)/18:0)	1.34	1.56	0.0022			
165.594	854.5684963	PC(22:5(4Z,7Z,10Z,13Z,16Z)/20:5(5Z,8Z,11Z,14Z,17Z))	1.21	0.034	1.86			
163.8115	878.5688202	PC(22:6(4Z,7Z,10Z,13Z,16Z,19Z)/22:6(4Z,7Z,10Z,13Z, 16Z,19Z))	1.28	2.66	0.0098			
166.5435	858.599035	PC(22:6(4Z,7Z,10Z,13Z,16Z,19Z)/20:2(11Z,14Z))	1.57	2.32	0.0035			
174.853	746.6063862	PC(18:0/P-16:0)	1.39	1.89	0.012			
166.493	862.6288863	PC(22:5(7Z,10Z,13Z,16Z,19Z)/20:1(11Z))	1.30	2.25	0.016			
62.0076	744.5905301	PC(18:1(11Z)/P-16:0)	1.29	0.021	1.57			
172.636	780.5527911	PC(18:4(6Z,9Z,12Z,15Z)/18:1(11Z))	1.21	0.5457	0.017			
175.336	718.5714715	PC(16:0/P-16:0)				1.39	0.64	0.033
38.34265	800.6150909	PC(22:2(13Z,16Z)/15:0)				1.66	0.67	0.013
174.4545	772.5832693	PC(20:2(11Z,14Z)/15:0)				1.55	0.77	0.032
179.737	706.5391278	PC(15:0/15:0)				1.66	0.56	0.021
217.669	880.5876355	PI(18:1(9Z)/18:1(9Z))				1.40	0.60	0.032
48.5458	722.5052151	PE(P-18:1(11Z)/18:4(6Z,9Z,12Z,15Z))				1.66	0.0023	0.52
220.325	572.3715597	LysoPC(22:4(7Z,10Z,13Z,16Z))	1.34	1.78	0.040			
218.54	578.4174168	LysoPC(22:1(13Z))	1.65	1.61	6.53E-05	1.63	0.83	0.017
217.282	580.433445	LysoPC(22:0)	1.29	1.49	0.016			
216.4165	506.3599418	LysoPC(P-18:1(9Z))	1.32	1.33	0.012			
230.847	468.3077333	LysoPC(14:0/0:0)				1.86	0.61	0.0011
275.518	476.2769555	LysoPE(18:3(6Z,9Z,12Z)/0:0)	1.44	3.01	0.0063			
228.222	502.2928886	LysoPE(20:4(5Z,8Z,11Z,14Z)/0:0)	1.43	2.32	0.0096			
231.697	478.2921673	LysoPE(0:0/18:2(9Z,12Z))	1.31	1.87	0.029			
211.7385	703.5747759	SM(d18:1/16:0)	1.59	1.61	0.00086	1.38	0.79	0.041
209.736	731.6042988	SM(d18:0/18:1(9Z))	1.43	1.83	0.0035			
206.061	799.667414	SM(d17:1/24:1(15Z))	1.10	0.66	0.035			
191.6825	729.5899261	SM(d18:1/18:1(9Z))	1.54	2.90	0.012			
214.093	675.5442252	SM(d18:1/14:0)	1.68	1.90	0.00044			
211.7385	703.5747759	SM(d18:1/16:0)				1.38	0.79	0.041
206.9425	785.6518627	SM(d16:1/24:1(15Z))				1.47	0.77	0.033
208.142	759.636123	SM(d18:1/20:0)				1.43	0.76	0.035
276.39	177.1023435	Serotonin	1.48	3.70	0.0014	1.66	0.33	0.0023
270.036	190.0866133	Indole-3-methyl aceta	1.62	4.59	4.65E-05	1.56	0.48	0.0089
164.0706936	25.8349	3-Methyldioxyindole	1.30	0.71	0.013			
200.595	190.0501312	Kynurenic acid	1.19	0.53	0.026			
276.262	160.0759615	Indoleacetaldehyde	1.44	4.66	0.00084	1.46	0.33	0.0025
287.167	118.0653429	Indole				1.80	0.66	0.0058
288.704	157.0760156	3-Indoleacetonitrile				1.44	0.63	0.032
290.47	192.0655478	5-Hydroxyindoleacetic acid				1.62	0.61	0.014

290.034	209.0920406	L-Kynurenine				1.75	0.54	0.0043
189.573	235.1076303	5-Methoxytryptophan	1.11	2.44	0.0020	1.45	0.54	0.017
46.84495	130.0651201	Indole-3-carbinol	1.28	0.63	0.037	1.53	1.41	0.020
47.7592	148.0391691	Indole-5,6-quinone	1.53	0.48	0.0040	1.40	1.93	0.040
107.5225	337.2374941	11,12-DHET	1.65	0.084	0.017	1.43	2.65	0.030
31.15675	303.2319237	8-HETE	1.25	1.72	0.022			
99.0816	333.2078313	Prostaglandin B2	1.48	0.36	0.020	1.78	2.23	0.0042
113.1645	335.2236599	Leukotriene B4	1.62	0.22	0.027	2.09	2.17	0.00031
55.70875	315.1965594	15-Deoxy-d-12,14-PGJ2	1.48	0.071	0.0061	2.09	44.78	0.0066
99.5398	317.2124862	5-KETE	1.78	0.22	0.011			
262.156	367.2131629	Prostaglandin G2	1.70	0.0077	0.029			
155.044	351.2180466	Prostaglandin D2	1.54	0.097	0.045			
66.4165	351.218008	Prostaglandin F3a	1.53	0.10	0.029			
199.394	353.2334763	8-Isoprostaglandin F2a				1.72	2.01	0.018
196.4295	351.2181516	Lipoxin B4				1.45	1.69	0.034
248.6275	180.065429	Hippuric acid				1.87	0.61	0.0024
326.777	136.0759321	2-Phenylacetamide				1.86	0.49	0.012
327.627	180.0661568	L-Tyrosine	1.67	0.54	0.017			
98.1862	137.0240068	3-Hydroxybenzoic acid	1.66	0.40	0.024	1.84	1.51	0.034
36.8827	147.0239485	3-Hydroxybenzoic acid	1.74	0.46	7.71E-05			
284.2975	164.0712597	L-Phenylalanine	1.19	0.78	0.050			

PC phosphatidylcholine, PI phosphatidyl inositol, PE phosphoethanolamine, PS phosphatidylserine, LysoPC lysophosphatidylcholine, LysoPE lysophosphoethanolamine, SM sphingomyelin, RT retention time, FC fold change, VIP variance importance for projection.
